# Post-Endovascular Abdominal Aortic Aneurysm Repair Abdominal Pain: A Learning Experience

**DOI:** 10.1177/2324709619865575

**Published:** 2019-07-26

**Authors:** Asim Kichloo, M. Zatmar Khan, El-Amir Zain, Navya Sree Vipparla, Farah Wani

**Affiliations:** 1 St. Mary’s Hospital, Saginaw, MI, USA; 2 Central Michigan University, Saginaw, MI, USA

**Keywords:** endovascular abdominal aortic aneurysm repair, abdominal aortic aneurysm

## Abstract

Abdominal aortic aneurysm (AAA) is one of the important pathologies involving the abdominal aorta, as it can have adverse consequences if it goes unnoticed or untreated. AAA is defined as an abnormal dilation of the abdominal aorta 3 cm or greater. Endovascular abdominal aortic aneurysm repair (EVAR) has recently emerged as a treatment modality for AAA. It does have a few inherent complications that include endoleak, endograft migration, bleeding, ischemia, and compartment syndrome. This case report discusses a patient who came in with abdominal pain and a pulsatile mass, which raised concerns regarding endoleak. The patient had a 9.9-cm AAA, which was repaired in the past, as was made evident by computed tomography findings of the stent graft in the aneurysmal segment. This case stands out because it highlights the importance of comparing the size of the AAA at the time of the EVAR to the current scenario where the patient presents with abdominal pain of unknown etiology. Also, this case report highlights the importance of computed tomography and other imaging forms in following-up with patients who have EVAR for AAAs.

## Introduction

Abdominal aortic aneurysm (AAA) is defined as an abnormal dilation of the abdominal aorta of at least more than 3 cm, with the average diameter of the normal abdominal aorta being about 2 cm.^1^ AAA carries an increased risk of rupture based on the size, with the risks associated with the following sizes: 1% to 3% per year for aneurysms between 4 and 5 cm, 6% to 11% per year for 5 to 7 cm, and 20% risk for greater than 7 cm.^2^ Treatment and management depends on patient preference and perioperative mortality and life expectancy, but the single most important factor that is considered in treatment and management is the diameter of the aneurysm.^2,3^

Endovascular abdominal aortic aneurysm repair (EVAR) has recently risen as a treatment of choice since being introduced by Parodi and colleagues in 1991.^2^ The procedure carries some complications, including endoleak, endograft migration, bleeding, ischemia, and compartment syndrome.^4^ Endoleak is the persistent flow of blood in the aneurysmal sac after the stent graft has been deployed; there are 5 classifications of endoleak, I to V.^2,5^ Type I endoleak results due to the loosening and possible detachment of either the proximal or distal anchors of the graft from the vessel wall. Type II is basically the back flow of blood into the aneurysmal sac from the aortic branch vessels. Types III and IV endoleaks share more or less the same causative factors: separation of the individual segments of the graft and increased porosity of the graft material, respectively. It is believed that increased pressure in the aneurysmal sac can result in Type V endoleak, sometimes referred to as endotension. This case report highlights the importance of understanding the complications of EVAR especially when the patients with prior history of EVAR presents with abdominal pain. It is imperative for the clinician to compare the size of AAA at the time of EVAR and follow-up AAA size to appropriately and promptly diagnose any life-threatening complication of EVAR.

## Case Report

**Figure 1.**
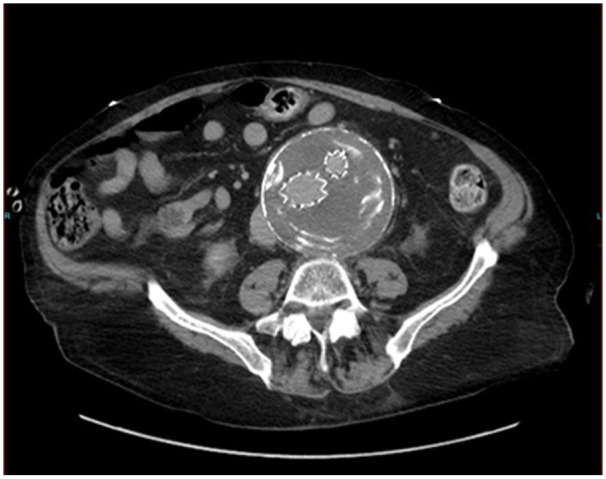
9.9-cm intra-AAA without any endoleak, with bilateral iliac limbs in place and laminated calcification of intramural thrombus.

**Figure 2.**
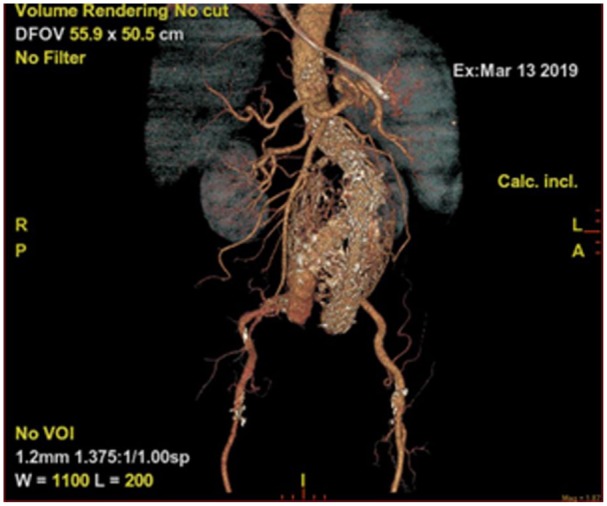
3D Reconstruction image of the Endovascular Aortic Graft.

A 71-year-old male presented to the emergency department with left lower quadrant and periumbilical abdominal pain for the past 3 days. He has had a history of AAA with endograft repair 3 years back. He denied any nausea, vomiting, or diarrhea. On presentation, he was normotensive and afebrile. Findings from the abdominal examination were notable for pulsatile mass in the left periumbilical area. Laboratory evaluation showed normal white blood cell count. Computed tomography (CT) scan of abdomen and pelvis initially without contrast showed endograft stent with kinking of the right iliac limb, which had possibly withdrawn from the iliac artery raising concerns for endograft failure (Type 1 endoleak) and a 9.9 cm infrarenal AAA. Computed tomographic angiogram (CTA) was recommended, which showed a consistent 9.9-cm intra-AAA without any endoleak, and both iliac limbs were in place with laminated calcification of intramural thrombus (Figures 1 and 2). CTA also revealed mild localized sigmoid diverticulitis. Reports from the vascular center where he had the surgery 3 years back reported the same size of aneurysm at the time of repair. He was managed conservatively as endoleak or expansion of the aneurysm size was ruled out. He followed-up in 1 month and was advised close vascular surgery follow-up.

## Discussion

AAA is one of the important pathologies involving the abdominal aorta, as it can have adverse consequences if it goes unnoticed or untreated. The prevalence of AAA is 7% to 8% in men older than the age of 65 years with smoking, age, ethnicity, hypercholesterolemia, and hypertension as the most common identified risk factors.^6^ Men older than the age of 65 years with a history of smoking should have a onetime screening done, but this age limit drops to 55 years if they have a family history of AAA.^7^ Physical examination findings hold importance in the diagnosis of AAA. In a study conducted by Chervu and colleagues, 38% of the 243 patients with AAA were diagnosed based on physical examination findings, while the other 62% on radiological imaging.^8^

First time surgical intervention is indicated in symptomatic patients in whom the size of the aneurysmal sac reaches about 5 to 5.5 cm in diameter.^5^ EVAR is one surgical option and is carried out using a stent graft, which consists of a nitinol framework surrounded by either polytetrafluoroethylene or polyester.^9^ This essentially focuses on correct placement of the graft and finding a favorable landing zone both proximally and distally.^4^ EVAR has gained widespread importance in the modern world because of shorter duration of hospital stay, lowered incidence of mechanical ventilation, and overall favorable outcomes.^10^ Important trials have been conducted on the outcomes associated with EVAR, namely, the EVAR-1 trial in the United Kingdom, ACE trial in France, DREAM trial in Netherlands, and OVER trial in the United States.^11^ In-hospital mortality rates in EVAR-1 and DREAM trials were 1.7% and 1.2% for EVAR as compared with 6% and 4.6% for open surgical repair, respectively.^7^ On a general note, it can be said that while EVAR has better short- and medium-term outcomes as compared with open surgical repair, there is no significant difference in the long-term outcomes.^12^

EVAR does have some complications, and endoleak is an important concern for patients with EVAR. While Types I and III are high-pressure lesions and require urgent intervention, Types II and IV can be managed conservatively and are generally due to a structural defect in the graft material.^4,7^ Eighty percent of Type II endoleaks resolve after 12 months and occur in 10% to 20% of the cases, while Type III (a high-pressure related endoleak) occurs in about 0% to 1.5% of cases.^5^ Our patient’s presentation that includes abdominal pain and finding of a pulsatile mass on physical examination made the most probable differential diagnosis as endoleak. The initial CT scan without contrast also raised further questions because of a withdrawn iliac limb. This was later ruled out when CTA was performed, which ruled out endoleak. This highlights the importance of CTA for ruling out endoleaks post-EVAR.

**Figure 3.**
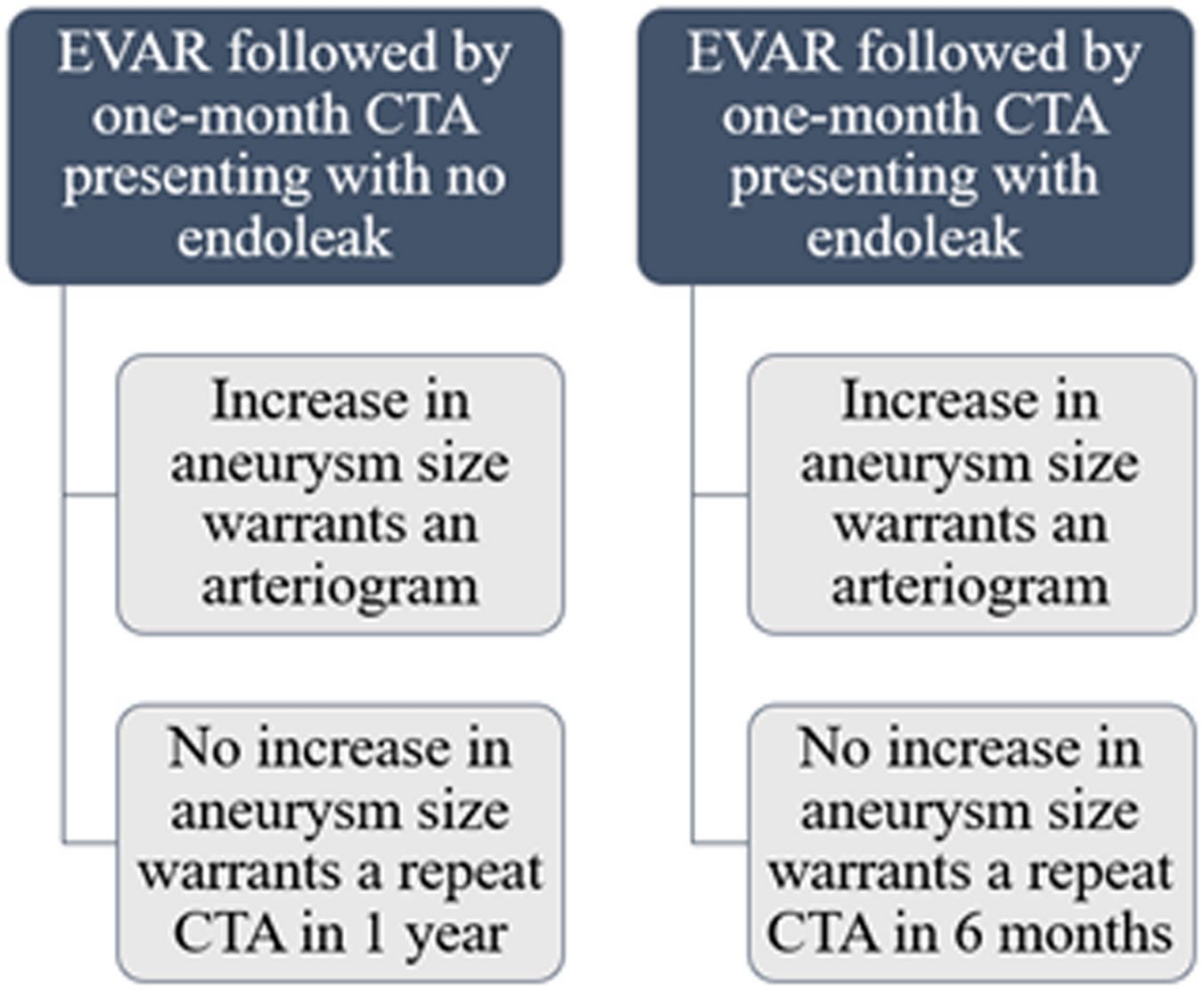
Recommended Post-EVAR follow-up.

Post-EVAR, patients need lifelong follow-ups (see Figure 3). Patient compliance with these follow-ups is reported to be about 50%.^13^ CTA has emerged as an important tool for follow-up assessments.^14^ The Society for Vascular Surgery recommends screening at 1- and 12-month periods following EVAR. The management of post-EVAR patients depends on 2 factors: endoleak and an increase in aneurysm size.^7^

Newer CT techniques have provided much insight into the detection of complications like endoleak, graft migration, and in-stent thrombosis; multiplanar and 3-dimensional reconstruction images are among the latest advances available at this time.^12^ For long-term follow-up, ultrasound is becoming popular due to decreased radiation exposure, no dye exposure, and cost-effectiveness.^15^

In this case, size of AAA at the time of EVAR and follow-up AAA size showed no increases in the sac size and CTA ruled out any endoleak.

## Conclusion

AAA is an important pathology in the field of vascular medicine and is now being treated more often using the EVAR. Complications are common and require undivided attention. The case presented here touched on AAA, EVAR, and endoleak, an important postoperative complication of EVAR. The importance of imaging techniques including CT scans and ultrasound cannot be overemphasized in the monitoring of these patients. Comparison to see the increase in the AAA size post-EVAR, along with CTA, the modality of choice, was helpful in excluding the diagnosis of endoleak.
